# The fibrosis-4 index is a prognostic factor for cholangiocarcinoma patients who received immunotherapy

**DOI:** 10.3389/fimmu.2024.1376590

**Published:** 2024-05-10

**Authors:** Zhiwei Zhang, Jingzhao Zhang, Ming Cai, Xiaorui Huang, Xinyi Guo, Dengsheng Zhu, Tong Guo, Yahong Yu

**Affiliations:** ^1^Department of Biliopancreatic Surgery, Tongji Hospital, Tongji Medical College, Huazhong University of Science and Technology, Wuhan, Hubei, China; ^2^Hubei Key Laboratory of Hepato-Biliary-Pancreatic Diseases, Wuhan, Hubei, China

**Keywords:** immunotherapy, immune checkpoint inhibitor, cholangiocarcinoma, biliary tract cancer, the fibrosis-4 index

## Abstract

**Background:**

Research of immunotherapy for cholangiocarcinoma has yielded some results, but more clinical data are needed to prove its efficacy and safety. Moreover, there is a need to identify accessible indexes for selecting patients who may benefit from such treatments.

**Methods:**

The medical records of 66 cholangiocarcinoma patients who underwent immunotherapy were retrospectively collected. The effectiveness of immunotherapy was assessed by tumor response, progression-free survival (PFS), and overall survival (OS), while safety was evaluated by adverse events during treatment. Univariate and multivariate Cox regression analyses were performed to identify prognostic risk factors for PFS and OS, and Kaplan-Meier curves of potential prognostic factors were drawn.

**Results:**

Overall, in this study, immunotherapy achieved an objective response rate of 24.2% and a disease control rate of 89.4% for the included patients. The median PFS was 445 days, and the median OS was 772.5 days. Of the 66 patients, 65 experienced adverse events during treatment, but none had severe consequences. Multivariate Cox analysis indicated that tumor number is a prognostic risk factor for disease progression following immunotherapy in cholangiocarcinoma patients, while tumor differentiation and the fibrosis-4 (FIB-4) index are independent risk factors for OS.

**Conclusion:**

In general, immunotherapy for cholangiocarcinoma is safe, with adverse events remaining within manageable limits, and it can effectively control disease progression in most patients. The FIB-4 index may reflect the potential benefit of immunotherapy for patients with cholangiocarcinoma.

## 1 Introduction

Cholangiocarcinoma is a highly malignant tumor with heterogeneity and can be categorized based on the location of occurrence into intrahepatic, perihilar, and distal cholangiocarcinoma. There is a considerable variation in the incidence rates across the world, with higher rates in China and other Asian countries (1). Moreover, the incidence is continually rising globally (2). To date, surgery remains the only potentially curative treatment for this disease. However, cholangiocarcinoma is characterized by insidious onset, rapid progression, and very poor prognosis. Often, it is diagnosed at a late stage when the surgical options are no longer viable. There is also a high tendency for recurrence or metastasis after surgery, further reducing the chances for additional surgical interventions. Therefore, the treatment of cholangiocarcinoma should involve a multidisciplinary approach centered around surgery, combined with systemic treatments such as chemotherapy, targeted therapy, and immunotherapy (3, 4).

The NCCN Guidelines for Biliary Tract Cancer recommend chemotherapy regimens with capecitabine, gemcitabine, oxaliplatin, cisplatin, and FOLFOX as systemic therapy (5). Compared to other types of cancer, such as non-small cell lung cancer, cholangiocarcinoma is relatively insensitive to immunotherapy, with research and application in this area progressing slowly. Fortunately, clinicians and researchers are striving to advance research on immunotherapy for bile duct cancer. Several studies have explored the effectiveness and safety of immune checkpoint inhibitors as monotherapy or in combination for patients with cholangiocarcinoma, from clinical trials to small-scale clinical applications. Studies on the application of pembrolizumab for incurable cholangiocarcinoma in Phase I b and II trials have found that regardless of PD-L1 expression, it can effectively control 6%-13% of advanced bile duct cancers, with manageable toxicity (6).A real-world study based on Phase III clinical trial TOPAZ-1, incorporating durvalumab into the first-line chemotherapy regimen for cholangiocarcinoma, demonstrated a 20% reduction in the risk of death (HR=0.80, 95% CI: 0.66-0.97, P=0.021), confirming the efficacy and safety of durvalumab for cholangiocarcinoma patients (7). Subsequently, more studies and case reports on the application of other immune checkpoint inhibitors in cholangiocarcinoma have emerged, further bolstering the case for immunotherapy in cholangiocarcinoma (8–11).Currently, the combination of durvalumab with gemcitabine and cisplatin is an option for first-line treatment of locally advanced or metastatic cholangiocarcinoma. However, further research is needed to verify the application of immune checkpoint inhibitors in cholangiocarcinoma and to identify prognostic risk factors for patients receiving immunotherapy.

The fibrosis-4 (FIB-4) index, incorporating age, ALT, AST, and platelet levels, is one of the most powerful indicators of liver fibrosis (12, 13). Studies have shown that FIB-4 can be used to predict the progression of extrahepatic tumors (14). Another study indicated that liver fibrosis could promote immune escape in hepatocellular carcinoma (15). Therefore, we hypothesize that FIB-4 can be a prognostic predictor of cholangiocarcinoma patients receiving immunotherapy. Inflammatory biomarkers such as the systemic immune-inflammation index (SII), neutrophil-to-lymphocyte ratio (NLR), and platelet-to-lymphocyte ratio (PLR) can reflect a patient’s systemic inflammatory and immune status (16, 17). Tumor-related inflammation is closely related to tumor occurrence, progression, immune responses, and patient prognosis (18–22). The calculation formula for SII is neutrophil count * platelet count/lymphocyte count. The prognostic nutritional index (PNI), which combines peripheral blood lymphocyte count and serum albumin level in a certain ratio, can be used to assess a patient’s nutritional and immune status (23) and is related to prognosis after receiving immunotherapy (24).

Therefore, the aim of this study is to explore the effectiveness and safety of immune checkpoint inhibitors in the treatment of cholangiocarcinoma and to investigate the predictive ability of the FIB-4 and inflammatory markers for patients with cholangiocarcinoma who received immunotherapy, providing further reference for application of immunotherapy in cholangiocarcinoma.

## 2 Materials and methods

### 2.1 Study design and participants

This study included a consecutive cohort of cholangiocarcinoma patients at Tongji Hospital affiliated with Huazhong University of Science and Technology from June 2018 to May 2022, including intrahepatic, perihilar, and extrahepatic cholangiocarcinoma. Pathological specimens obtained through surgery or biopsy were confirmed by expert pathologists. Inclusion criteria were as follows: (1) undergoing more than one cycles of immunotherapy; (2) immune checkpoint inhibitors were not used in the perioperative period; (3) no history of other malignancies; (4) complete data. All data were collected retrospectively from medical records, including general data, laboratory tests, and imaging data within 7 days before the first immunotherapy. The end point of follow-up was the patient’s death, loss to follow-up, or survival at the end of the follow-up period, which was December 31, 2023. This research was approved by the Research Ethics Committee of Tongji Hospital affiliated with Huazhong University of Science and Technology.

### 2.2 Response assessment

Tumor response was assessed 4-6 weeks after the second immunotherapy by CT or MRI, compared with imaging data within one week prior to the first immunotherapy and reported by expert radiologists. The criteria for assessment were based on RECIST 1.1 (25). The results included immune complete response (iCR), immune partial response (iPR), immune stable disease (iSD), and immune progressive disease (iPD). Additional evaluation metrics were the objective response rate (ORR) and disease control rate (DCR). ORR was the sum of iCR and iPR, and DCR was the sum of iCR, iPR, and iSD.

### 2.3 Safety evaluation

Patients’ immune-related adverse events (irAEs) during treatment with immune checkpoint inhibitors were evaluated for the safety of immunotherapy through retrospective review of medical records, including blood routine, liver function, renal function, thyroid function, and recorded symptoms such as weight loss, fatigue, decreased appetite, rash, etc.

### 2.4 Statistical analysis

The presentation of data is described in the tables. Continuous variables were presented as median (interquartile range). Categorical variables were presented as numbers (percentages). The cutoff values for continuous variables were the critical values of the test items or obtained through the R package surv_cutpoint based on the time and status of overall survival (OS). Risk factors for progression-free survival (PFS) or OS were analyzed using a Cox regression model, and a P-value of less than 0.05 was considered statistically significant. Survival curves were generated using the Kaplan-Meier (K-M) method through the survminer package in R, with p-values calculated via the log-rank test. The K-M curves and adverse events were visualized using the ggplot2 package. Analysis involving the specified R packages was conducted using R 4.3.2, while sections not mentioned to use R packages were analyzed using SPSS 26.

## 3 Results

### 3.1 Baseline characteristics of patients

**Figure 1:**
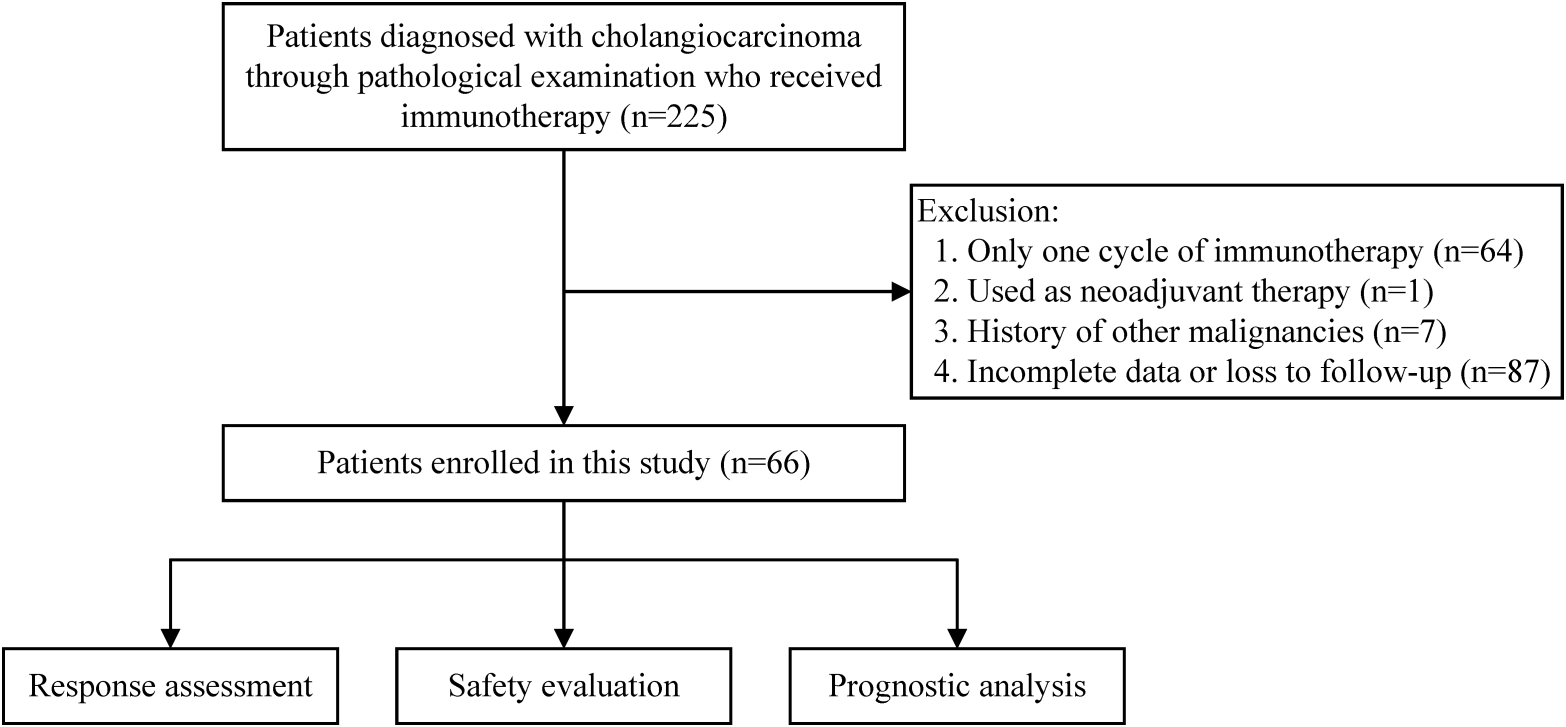
The flowchart of this study.

Between June 1, 2018, and May 31, 2022, a total of 225 cholangiocarcinoma patients receiving immunotherapy were registered at our center. These patients all underwent surgical resection or percutaneous biopsy to obtain tumor tissue and were diagnosed with cholangiocarcinoma following pathological examination. After excluding patients who only received one cycle of immunotherapy, those for whom immunotherapy was used as neoadjuvant therapy, those with a history of other malignancies, those with incomplete case data or who were lost to follow-up, 66 patients were ultimately included in this study. The number of patients excluded for each criterion is shown in the flowchart (**Figure 1**).

The study follow-up was conducted up until December 31, 2023, with the shortest follow-up duration being 18 months for the included patients. All patients had an ECOG score of either 0 or 1. The study population comprised 37 (56.1%) males and 29 (43.9%) females, with an age distribution of 29-79 years and a median age of 57.5 years. Based on the originating site within the biliary system, there were 57 cases (86.4%) of intrahepatic cholangiocarcinoma, 7 cases (10.6%) of perihilar cholangiocarcinoma, and 2 cases (3.0%) of distal cholangiocarcinoma. Among them, 21 patients had a history of hepatitis B virus infection, accounting for 31.8% of the total. At the time of immunotherapy, more than half of the patients (59.1%) had carbohydrate antigen 19-9 (CA19-9) levels beyond the normal range, and a small portion of patients (26.9%) had abnormal carcinoembryonic antigen (CEA) levels. Approximately half of the patients (45.5%) had abnormal liver enzymes with or without increased bilirubin levels. The vast majority of patients had adenocarcinoma pathology (95.5%). Among all patients, only one case was highly differentiated, with the rest being poorly differentiated (25.8%) or moderately differentiated (72.7%). Immunohistochemistry results showed a median Ki67 value of 40, with quartiles of 15 and 69. Those included in this study for immunotherapy were all at an advanced stage of the disease, with a median tumor largest diameter of 4.8cm. 39 cases (59.1%) had multiple lesions, 10 cases (15.2%) had major vascular invasion, 33 cases (50%) local lymph node metastasis, 36 cases (54.5%) liver metastasis, and 14 cases (21.2%) distant metastasis beyond the liver. The baseline characteristics are shown in **Table 1**.

**Table 1:** Baseline characteristics.

Variables	Total (n=66)
Age, years, median (range)	57.5 (29.0-79.0)
Sex (male)	37 (56.1)
BMI	
<18.5	1 (1.5)
18.5-24	51 (77.3)
>24	14 (21.2)
History of HBV infection	21 (31.8)
KPS	
50	1 (1.5)
60	3 (4.5)
70	12 (18.2)
80	49 (74.2)
90	1 (1.5)
CEA, ng/mL (<5)	49 (73.1)
CA19-9, U/mL (<34)	27 (40.9)
Lymphocyte, 10^9/L, median (quartile)	1.30 (0.98-1.78)
Neutrophil, 10^9/L, median (quartile)	3.59 (2.54-5.03)
Platelet, 10^9/L, median (quartile)	190.50 (150.00-263.75)
ALT, U/L, (<33)	44 (66.7)
AST, U/L, (<32)	40 (60.6)
ALB, g/L, (<35)	7 (10.6)
LDH, U/L, (<214)	44 (66.7)
TBil,μmol/L, (<21)	58 (87.9)
FIB-4, median (quartile)	1.72 (1.26-2.26)
SII, median (quartile)	472.34 (329.15-885.45)
NLR, median (quartile)	2.60 (1.80-4.11)
PLR, median (quartile)	137.08 (105.18-190.33)
PNI, median (quartile)	46.50 (43.45-51.13)
Child-Pugh stage	
A	62 (93.9)
B	4 (6.1)
Histological type	
Adenocarcinoma	63 (95.5)
Other types	3 (4.5)
Differentiation	
Well	1 (1.5)
Medium	48 (72.7)
Poor	17 (25.8)
Ki67, median (quartile)	40 (15-69)
Tumor number	
Single	27 (40.9)
Multiple	39 (59.1)
Tumor site	
Intrahepatic	57 (86.4)
Perihilar	7 (10.6)
Distal	2 (3.0)
Largest tumor diameter, cm, median (quartile)	4.8 (2.8-7.1)
Macrovascular invasion	10 (15.2)
Local lymph node metastasis	33 (50.0)
Liver metastasis	36 (54.5)
Extrahepatic metastasis	
Omentum majus	2 (3.0)
Lung	3 (4.5)
Brain	1 (1.5)
Stomach	1 (1.5)
Supraclavicular lymph nodes	2 (3.0)
Bone	2 (3.0)
Pancreas	3 (4.5)

Values are presented as median (quartile) or n (%).

BMI, body mass index; HBV, hepatitis B virus; KPS, Karnofsky performance status score; CEA, carcinoembryonic antigen; CA19-9, carbohydrate antigen 19-9; ALT, alanine transaminase; AST, aspertate aminotransferase; ALB, albumin; LDH, lactate dehydrogenase; Tbil, total bilirubin; FIB-4, the fibrosis-4 index; SII, systemic immune-inflammation index; NLR, neutrophil-to-lymphocyte ratio; PLR, platelet-to-lymphocyte ratio; PNI, prognostic nutritional index.

### 3.2 Treatment strategy for immunotherapy

The treatment strategy is displayed in **Table 2**. In this study, the median number of continuous immunotherapy sessions was 4.5, with one individual undergoing up to 27 sessions. Most patients had previously undergone various treatments before receiving immunotherapy, such as surgery, TACE, microwave ablation, radiotherapy, chemotherapy, or combinations of these treatments. 21 patients used immunotherapy as a first-line treatment; among them, a few employed only immunotherapy as the first-line treatment, often combined with chemotherapy or targeted therapy or both simultaneously. Among all cases included in this study, during the immunotherapy phase, 13 individuals (19.7%) applied immunotherapy alone, around half of the patients (n=34, 51.5%) combined with chemotherapy, and 11 patients (12.1) combined with targeted therapy, with 8 patients receiving a combination of chemotherapy, immunotherapy, and targeted therapy.

**Table 2:** Treatment strategy.

Variables	Total (n=66)
Sequence of immunotherapy	
First-line	45 (68.2)
Second-line	21 (31.8)
Treatment prior to immunotherapy	
Surgery	36 (54.5)
TACE	14 (21.2)
Microwave ablation	4 (6.1)
Radiotherapy	11 (16.7)
Chemotherapy	48 (72.7)
Treatment combined with immunotherapy	
Immunotarapy alone	13 (19.7)
Combined with chemotherapy	34 (51.5)
Combined with targeted therapy	11 (12.1)
Combined with chemotherapy and targeted therapy	8 (12.1)
Total course of immunotherapy, median (range)	4.5 (2-27)

Values are presented as n (%).

TACE, transarterial chemoembolization.

### 3.3 Efficacy

In this study, a total of eight different immune checkpoint inhibitors were used, with Camrelizumab being the most frequently employed. No cases were rated as having achieved iCR. Overall, the ORR post-immunotherapy was 24.2%, and the DCR was 89.4%. Specific to each inhibitor, Durvalumab had the highest DCR of 100%. Following up were Camrelizumab and Tislelizumab with DCRs of 91.3% and 90.9%, respectively. Toripalimab had the lowest ORR (12.5%) and DCR (75.0%). In the entire cohort, the median overall survival was 772.5 days, and the median progression-free survival was 445 days. Information was detailed in **Table 3**.

**Table 3:** Tumor response, PFS and OS.

	Total (n=66)	Durvalumab (n=9)	Camrelizumab (n=23)	Nivolumab (n=1)	Pembrolizumab (n=7)	Toripalimab (n=8)	Tislelizumab (n=7)	Sintilimab (n=11)
Tumor response								
iCR	0 (0)	0 (0)	0 (0)	0 (0)	0 (0)	0 (0)	0 (0)	0 (0)
iPR	16 (24.2)	2 (22.2)	6 (26.1)	0 (0)	2 (28.6)	1 (12.5)	2 (28.6)	3 (27.3)
iSD	43 (65.2)	7 (77.8)	15 (65.2)	1 (100)	4 (57.1)	5 (62.5)	4 (57.1)	7 (62.6)
iPD	7 (10.6)	0 (0)	2 (8.7)	0 (0)	1 (14.3)	2 (25.0)	1(14.3)	1 (9.1)
ORR	16 (24.2)	2 (22.2)	6 (26.1)	0 (0)	2 (28.6)	1 (12.5)	2 (28.6)	3 (27.3)
DCR	59 (89.4)	9 (100.0)	21 (91.3)	1 (100.0)	6 (85.7)	6 (75.0)	6 (85.7)	10 (90.9)
PFS, median(quartile), days	445 (192.3-926.0)							
OS, median(quartile), days	772.5 (399.3-1069.0)							

Values are presented as n (%).

iCR, immune complete response; iPR, immune partial response iPR; iSD, immune stable disease; iPD, immune progressive disease; ORR, objective response rate; DCR, disease control rate; PFS, progression-free survival; OS, overall survival.

### 3.4 Safety

**Figure 2:**
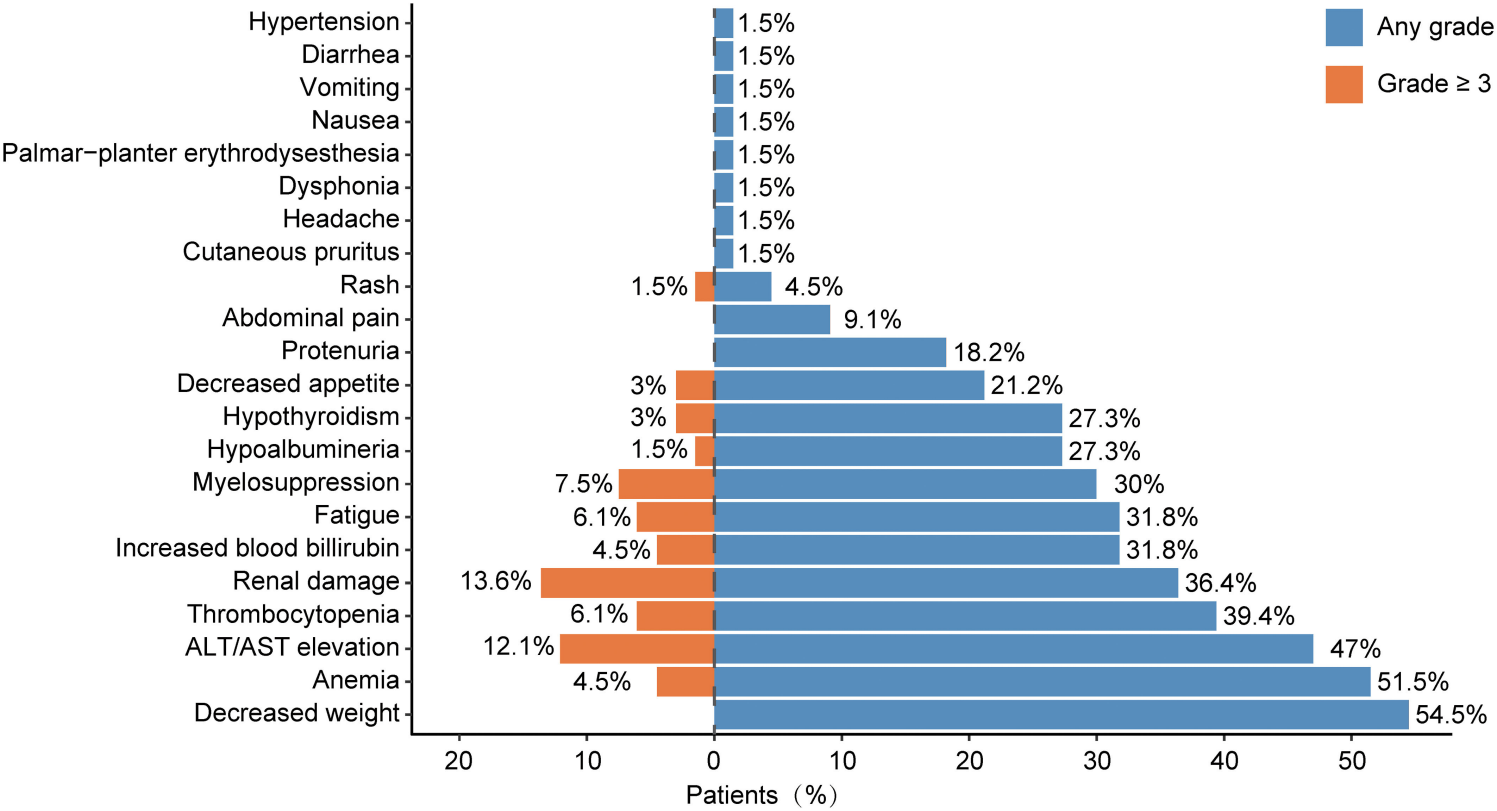
Occurrence rate of adverse events in this study.

The safety of immunotherapy for cholangiocarcinoma was assessed by evaluating immune-related adverse events (irAEs). Of the 66 cholangiocarcinoma patients included in the study, 65 experienced at least one type of adverse reaction during immunotherapy. All recorded adverse reactions are displayed in **Figure 2**. The most frequently recorded adverse event was weight loss. Other adverse events occurring in over 5% of patients included anemia (51.5%), ALT/AST elevation (47%), thrombocytopenia (39.4%), renal damage (36.4%), increased blood bilirubin (31.8%), fatigue (31.8%), myelosuppression (30.0%), hypoalbuminemia (27.3%), hypothyroidism (27.3%), decreased appetite (21.2%), proteinuria (18.2%), and abdominal pain (9.1%). Grade 3 or higher irAEs that were relatively common included renal damage (13.6%), ALT/AST elevation (12.1%), myelosuppression (7.5%), and thrombocytopenia (6.1%).

### 3.5 Risk factors

The study subsequently explored risk factors for survival and progression among the enrolled patients. After a comprehensive evaluation, 26 potential prognostic indicators were included for Cox regression analysis of OS and PFS, with results presented in **Tables 4**, **5**. Body mass index (BMI) values were categorized into three groups based on international definitions for underweight, normal, and overweight, using 18.5 and 24.0 as cut-off points. Karnofsky performance status (KPS) score, Ki67, and the total course of immunotherapy were analyzed as continuous variables. Due to only one case of high differentiation, well-differentiated and moderately-differentiated cases were pooled for comparison analysis against poorly-differentiated cases. CEA, CA19-9, albumin (ALB), lactate dehydrogenase (LDH), and total bilirubin (TBil) were divided into two groups based on normal value boundaries of these tests. Optimal cut-off values for FIB-4, SII, NLR, PLR, and PNI were calculated using the R package “surv_cutpoint,” and used to divide patients into two groups for subsequent analyses. Based on the results of univariate analyses, only tumor number (Multiple vs. Single, P=0.015, HR=2.285, 95%CI:1.172-4.454) was statistically significant for PFS; for OS, the following nine variables were found to be statistically significant: KPS (P=0.03, HR=0.944, 95%CI:0.897-0.994), CA19-9 (≥5 vs. < 5 U/mL, P=0.045, HR=2.172, 95%CI:1.018-4.637), TBil (≥21 vs.<21 μmol/L, P=0.004, HR=4.302, 95%CI:1.6-11.569), Differentiation (Poor vs. Well + Medium, P=0.002, HR=3.079, 95%CI:1.429-6.353), Tumor number (Multiple vs. Single, P=0.031, HR=2.557, 95%CI:1.090-5.999), FIB-4 (≥3.29 vs.<3.29, P=0.023, HR=2.686, 95%CI:1.447-6.287), SII (≥1165.96 vs.<1165.96, P=0.004, HR=3.505, 95%CI:1.505-8.167), PLR (≥296.72 vs.<296.72, P=0.033, HR=2.739, 95%CI:1.084-6.923), PNI (≥43.60 vs.<43.60, P=0.011, HR=0.382, 95%CI:0.183-0.800). These nine variables were incorporated into the multivariate analysis with OS as the outcome variable. For the PFS outcome, CA19-9 and tumor number were entered into further multivariate analysis. Eventually, tumor number (Multiple vs. Single, P=0.034, HR=2.083, 95%CI:1.056-4.108) was identified as an independent risk factor for PFS. For OS, tumor differentiation (Poor vs. Well + Medium, P=0.007, HR=3.534, 95%CI:1.405-8.886) and FIB-4 (≥3.29 vs.<3.29, P=0.031, HR=3.219, 95%CI:1.112-9.319) were prognostic risk factors.

**Table 4:** Univariate and multivariate analysis of OS.

Variables	Univariate			Multivariate		
	HR	95% CI	P	HR	95% CI	P
Age (≥60/<60)	0.845	0.419-1.704	0.638			
Sex (male/female)	1.335	0.664-2.683	0.418			
BMI						
18.5-24	Ref.					
<18.5	5.349	0.684-41.806	0.110			
>24	0.966	0.420-2.222	0.934			
History of HBV infection (Yes/No)	0.751	0.360-1.568	0.446			
KPS	0.944	0.897-0.994	0.030	0.946	1.405-8.886	0.088
CEA (≥5/<5)	1.224	0.542-2.768	0.627			
CA19-9 (≥34/<34)	2.172	1.018-4.637	0.045	2.493	0.988-6.294	0.053
ALB (≥35/<35)	0.788	0.275-2.264	0.658			
LDH (≥214/<214)	1.707	0.820-3.556	0.153			
TBil (≥21/<21)	4.302	1.600-11.569	0.004	1.265	0.229-6.984	0.787
Child-Pugh stage (B/A)	2.214	0.522-9.385	0.281			
Histological type (Other types/Adenocarcinoma)	1.020	0.241-4.324	0.978			
Differentiation (Poor/Well+Medium)	3.079	1.429-6.353	0.002	3.534	1.405-8.886	0.007
Ki67	1.009	0.994-1.025	0.246			
Tumor number (Multiple/Single)	2.557	1.090-5.999	0.031	2.091	0.768-5.688	0.149
Timor site						
Intrahepatic	Ref.					
Perihilar	1.457	0.436-4.866	0.541			
Distal	1.357	0.316-5.835	0.681			
Largest tumor diameter	0.973	0.875-1.082	0.616			
Macrovascular invasion (Yes/No)	0.894	0.268-2.979	0.855			
Local lymph node metastasis (Yes/No)	1.006	0.495-2.048	0.986			
Liver metastasis (Yes/No)	1.162	0.554-2.437	0.692			
FIB-4 (≥3.29/<3.29)	2.686	1.147-6.287	0.023	3.219	1.112-9.319	0.031
SII (≥1165.96/<1165.96)	3.505	1.505-8.167	0.004	3.501	0.825-14.849	0.089
NLR (≥6.04/<6.04)	2.001	0.810-4.945	0.133			
PLR (≥296.72/<296.72)	2.739	1.084-6.923	0.033	1.005	0.298-3.389	0.994
PNI (≥43.60/<43.60)	0.382	0.183-0.800	0.011	0.592	0.205-1.707	0.332
Total course of immunotherapy	0.939	0.866-1.018	0.126			

P-value < 0.05 is statistically significant.

OS, overall survival; HR, hazard ratio; CI, confidence interval; Ref, reference; BMI, body mass index; HBV, hepatitis B virus; KPS, Karnofsky performance status score; CEA, carcinoembryonic antigen; CA19-9, carbohydrate antigen 19-9; ALB, albumin; LDH, lactate dehydrogenase; Tbil, total bilirubin; FIB-4, the fibrosis-4 index; SII, systemic immune-inflammation index; NLR, neutrophil-to-lymphocyte ratio; PLR, platelet-to-lymphocyte ratio; PNI, prognostic nutritional index.

**Table 5:** Univariate and multivariate analysis of PFS.

Variables	Univariate			Multivariate		
	HR	95% CI	P	HR	95% CI	P
Age (≥60/<60)	0.805	0.421-1.539	0.512			
Sex (male/female)	0.704	0.372-1.334	0.282			
BMI						
18.5-24	Ref.					
<18.5	7.003	0.879-55.827	0.066			
>24	1.248	0.608-2.564	0.546			
History of HBV (Yes/No)	1.149	0.605-2.184	0.671			
KPS	0.962	0.910-1.017	0.176			
CEA (≥5/<5)	1.182	0.599-2.332	0.630			
CA19-9 (≥34/<34)	1.894	0.997-3.598	0.051	1.662	0.866-3.189	0.126
ALB (≥35/<35)	1.214	0.431-3.424	0.714			
LDH (≥214/<214)	1.083	0.559-2.098	0.812			
TBil (≥21/<21)	2.040	0.783-5.319	0.145			
Child-Pugh stage (B/A)	1.123	0.269-4.688	0.874			
Histological type (Other types/Adenocarcinoma)	0.747	1.179-3.113	0.688			
Differentiation (Poor/Well+Medium)	1.740	0.873-3.467	0.115			
Ki67	0.998	0.984-1.011	0.720			
Tumor number (Multiple/Single)	2.285	1.172-4.454	0.015	2.083	1.056-4.108	0.034
Tumor site						
Intrahepatic	Ref.					
Perihilar	1.815	0.753-4.372	0.184			
Distal	1.084	0.258-4.560	0.912			
Largest tumor diameter	0.951	0.865-1.046	0.302			
Macrovascular invasion (Yes/No)	0.635	0.225-1.791	0.391			
Local lymph node metastasis (Yes/No)	0.986	0.530-1.834	0.965			
Liver metastasis (Yes/No)	1.326	0.707-2.485	0.379			
FIB-4 (≥3.36/<3.36)	1.544	0.597-3.991	0.370			
SII (≥929.98/<929.98)	1.254	0.522-3.012	0.613			
NLR (≥6.34/<6.34)	0.997	0.386-2.575	0.994			
PLR (≥207/<207)	1.112	0.435-2.885	0.814			
PNI (≥43.675/<43.675)	0.812	0.403-1.634	0.559			
Total course of immunotherapy	0.974	0.914-1.039	0.424			

P-value < 0.05 is statistically significant.

PFS, progression-free survival; HR, hazard ratio; CI, confidence interval; Ref, reference; BMI, body mass index; HBV, hepatitis B virus; KPS, Karnofsky performance status score; CEA, carcinoembryonic antigen; CA19-9, carbohydrate antigen 19-9;ALB, albumin; LDH, lactate dehydrogenase; Tbil, total bilirubin; FIB-4, the fibrosis-4 index; SII, systemic immune-inflammation index; NLR, neutrophil-to-lymphocyte ratio; PLR, platelet-to-lymphocyte ratio; PNI, prognostic nutritional index.

**Figure 3:**
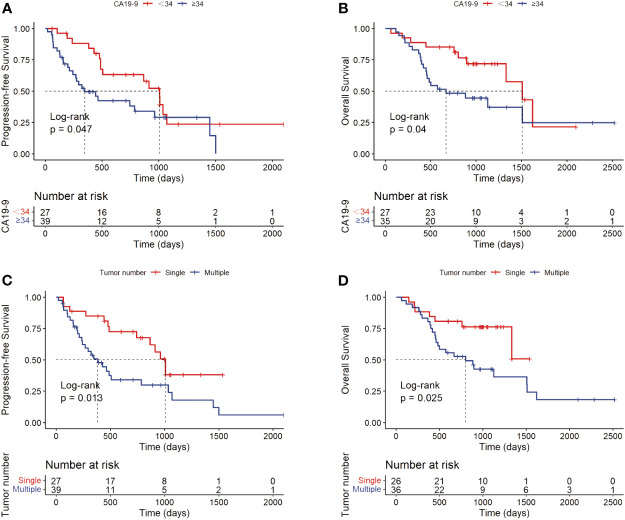
Kaplan-Meier curves of CA19-9 (**A**: PFS; **B**: OS) and tumor number (**C**: PFS; **D**: OS).

**Figure 4:**
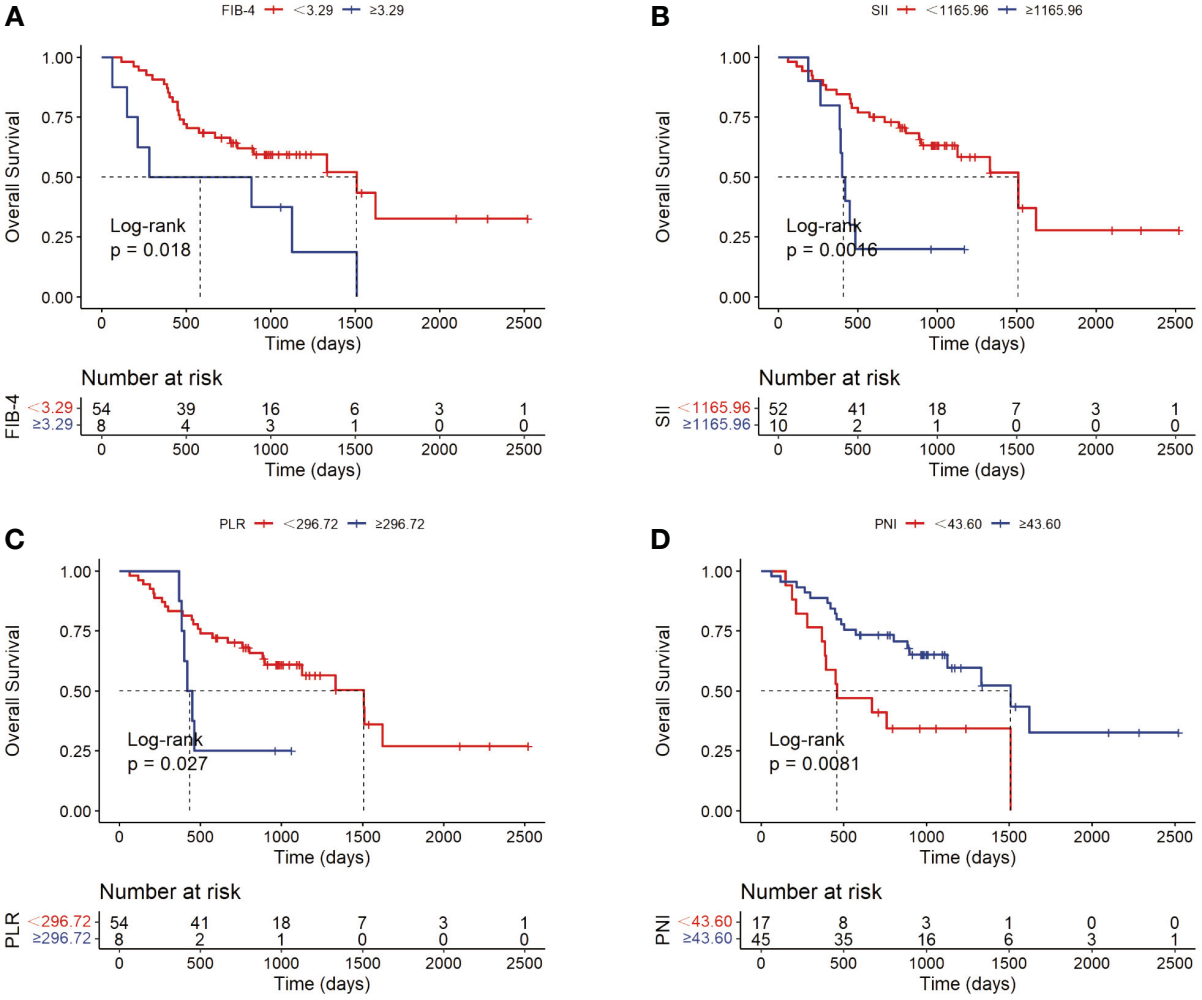
Kaplan-Meier analyses of FIB-4 **(A)**, SII **(B)**, PLR **(C)** and PNI **(D)** with OS as the outcome variable.

For potential risk factors identified by univariate analysis, K-M curves were plotted according to cut-off values, and P-values were calculated between different groups using the log-rank test. As shown in **Figure 3**, patients with higher levels of CA19-9 at the start of immunotherapy had a higher risk of tumor progression and shorter survival, with median PFS (mPFS) of 1007 vs. 348 days, and median OS (mOS) of 1509 vs. 669 days. Patients with multiple tumors had significantly shorter progression times than those with a single tumor (mOS: 1007 vs. 383 days), and notably shorter survival times, with half of single-tumor patients surviving at the conclusion of follow-up. **Figure 4** shows the impact of FIB-4, SII, PLR, and PNI on OS. Patients with higher levels of FIB-4, SII, and PLR had shorter OS, while those with higher PNI levels had longer OS. K-M curves of NLR and TBil are shown in **Supplementary Figure S1**.

## 4 Discussion

Recent years have seen a burgeoning interest in clinical and basic research on immune checkpoint inhibitors, which have shown promising results in a variety of cancers, including some progress in cholangiocarcinoma. The combination of durvalumab with gemcitabine and cisplatin as a first-line treatment for cholangiocarcinoma has been approved as an indication for durvalumab. Additional clinical studies using immune checkpoint inhibitors as a first-line treatment for cholangiocarcinoma are underway (26, 27). At present, before immunotherapy, the most effective method for assessing possible tumor response and predicting the efficacy of immunotherapy is to biopsy the tumor tissue for PD-L1 immunohistochemistry staining. The tumor proportion score (TPS), immune cell score (IC), and tumor burden score (TBS) can be calculated based on the staining results to assess PD-L1 expression (28). However, PD-L1 testing is not routinely available in many hospitals, and patient acceptance is not high. For those diagnosed with a needle or aspiration biopsy, the sample volume is limited and may not be sufficient for PD-L1 staining. Therefore, we need simple and effective indicators to predict the prognosis of cholangiocarcinoma patients undergoing immunotherapy. This study explores the potential of comprehensive indicators such as FIB-4, SII, NLR, PLR, PNI based on various simple blood test results to predict patient prognosis.

A total of 66 patients with pathological results and who had received immunotherapy were included in this study. Most were in the advanced stages of the disease at the time of immunotherapy, with multiple lesions or macrovascular invasion, or distant metastasis. The mOS in the cohort was 772.5 days, and the mPFS was 445 days, with an DCR of 89.4%, congruent with previous studies (29). Among the 66 patients, only one did not experience any adverse events, with decreased weight being the most commonly reported side effect. In patients who developed irAEs, only eight individuals (12.1%) experienced a single adverse event, while the remainder had at least two types of irAEs. Adverse reactions affected the immunotherapy regimen in 33 patients, accounting for 50.7% of the cohort, with renal damage being the most common severe (grade 3 or higher) adverse event. Notably, one patient receiving sintilimab discontinued treatment due to severe bone marrow suppression (grade 4). According to a multicenter study that included 53 patients, researchers reported that all patients experienced irAEs (30). Fatigue, both in any grade and grade 3 or higher, was the most common adverse event, with 41.5% of patients experiencing severe (grade 3 or higher) reactions, and one case of grade 4 bone marrow suppression, findings similar to those of our study. Another study (31) divided 59 cholangiocarcinoma patients who underwent immunotherapy into two groups based on the occurrence of irAEs (32 in the irAE group, accounting for 54.2%) and compared differences between them. They observed statistical differences in total bilirubin levels and relapse between the two groups. Through Cox regression analysis, an interesting conclusion was drawn—whether irAEs occurred was an independent risk factor for the OS and PFS of patients with advanced cholangiocarcinoma. Our research, along with these studies, underscores the necessity of investigating irAEs in cholangiocarcinoma patients, as it is meaningful for assessing the safety and efficacy of immunotherapy.

To explore prognostic risk factors, univariate and multivariate Cox regression analyses were conducted with PFS and OS as outcome variables, and the results of the univariate analysis showing statistical significance were subjected to log-rank testing. We found that patients with multiple tumors before immunotherapy had a worse prognosis, more likely to show progression after immunotherapy treatment (mOS: 1007 vs. 383 days), with a significantly shorter overall survival. A meta-analysis based on 47 research cohorts suggested tumor number as a predictive factor in hepatocellular carcinoma OS and PFS predictive models (32). Kaplan-Meier analyses revealed that, in this study, the traditional tumor marker CA19-9 has good short-term predictive power for OS and PFS in patients with cholangiocarcinoma. A Chinese study on pancreatic cancer patients undergoing immunotherapy found that higher baseline levels of CA19-9 were associated with accelerated tumor growth during immunotherapy and worse prognosis (33). Furthermore, poorly differentiated tumors were found to grow rapidly, to be more invasive, more likely to metastasize, and had significantly worse prognosis (mOS: 669 vs. 1509 days), which was an independent risk factor predicting the outcome of immunotherapy for cholangiocarcinoma. Obstruction often occurs in biliary diseases, leading to elevated serum liver enzyme and bilirubin levels. In our study, bilirubin levels within a week before receiving immunotherapy were related to the total survival time of the patients (mOS: 1508 vs. 263 days), with higher levels indicating a worse prognosis. Bilirubin levels affect patients with cholangiocarcinoma’s prognosis in several ways: Patients with hilar cholangiocarcinoma with higher bilirubin levels at diagnosis may not benefit from surgery (34); The albumin bilirubin (ALBI) grade, which combines bilirubin and albumin levels, is an independent prognostic factor for OS and PFS in intrahepatic cholangiocarcinoma (35, 36).

The analysis of FIB-4, SII, NLR, PLR, and PNI showed that FIB-4 is an independent risk factor for OS in patients with cholangiocarcinoma undergoing immunotherapy. Initially identified as a non-invasive marker for predicting and diagnosing liver fibrosis, FIB-4 quickly gained widespread application and holds significant value in various diseases (37, 38). Its most extensively studied aspect is the correlation between FIB-4 and the incidence, progression, and prognosis of liver cancer, as well as the response to treatment. Studies have shown that FIB-4 can predict the recurrence of hepatocellular carcinoma following curative hepatectomy (39). Large sample size researches in multiple countries have explored FIB-4 in relation to various diseases. For instance, a study in Germany found that elevated FIB-4 levels are associated with an increased risk of developing liver cancer (40, 41). In the United States, a prospective study using FIB-4 as one of the markers for predicting liver fibrosis found that higher fibrosis levels were linked to an increased overall mortality rate from liver diseases (42). A retrospective study in Korea revealed that individuals with high FIB-4 levels had a significantly increased risk of dying from liver cancer (HR: 629.10, 95% CI: 228.74-1730.20) (43). Moreover, FIB-4’s predictive value for the likelihood of hepatocellular carcinoma in moderate to heavy drinkers even surpasses that of ultrasound-detected liver cirrhosis indices (44). For individuals with HCV infection, FIB-4 levels are closely associated with the risk of developing liver cancer after achieving HCV clearance (45), and therapeutic interventions to reduce FIB-4 levels can decrease the risk of hepatocellular carcinoma in these patients (46, 47). FIB-4 is also closely related to the prognosis of various extrahepatic tumors (48). A large cohort study in Korea involving 25,947 individuals explored the relationship between non-alcoholic fatty liver disease and various types of cancer, finding a strong correlation between high FIB-4 levels and the incidence of all types of tumors (49). In colorectal cancer patients, high pre-operative or pre-chemotherapy FIB-4 levels could reliably predict poorer long-term outcomes post-surgery (50), and patients with high FIB-4 levels had a greater likelihood of synchronous liver metastasis from colorectal cancer (51). Additionally, FIB-4 can predict the long-term prognosis after gastric cancer resection (52). FIB-4’s correlation extends to mortality from non-tumorous diseases as well. Another large study in Korea found that an FIB-4 level >2.67 was associated with increased all-cause mortality, cardiovascular mortality, and liver-related mortality (53). This index could even predict the mortality rate in COVID-19 patients (54), with an FIB-4 score >2.53 being related to an increased risk of death (OR: 4.53, 95% CI: 2.83-7.25, P<0.001) (55). In this study, higher levels of FIB-4 were associated with a worse prognosis after treatment with immune checkpoint inhibitors. Research suggests that liver fibrosis can facilitate the immune escape of hepatocellular carcinoma (15). Given the proximity of cholangiocarcinoma and hepatocellular carcinoma development sites, this might partially explain the poorer immune treatment responses in patients with elevated FIB-4 levels in our study. However, NLR levels were not significantly correlated with recurrence or prognosis. Higher levels of SII and PLR and lower levels of PNI were associated with a worse prognosis, consistent with previously published research findings (56–59).

Our study has limitations as PD-L1 expression was not routinely measured at our center. Consequently, we could not discuss the relationship between PD-L1 expression and immune response, progression, or prognosis in cholangiocarcinoma patients. Moreover, as this is a single-center, small sample, retrospective study, information bias cannot be completely avoided, and larger and prospective studies are needed to confirm the causal relationship between FIB-4 levels and prognosis in cholangiocarcinoma patients undergoing immunotherapy. Besides, this study did not include a comparison between patients who only received chemotherapy and those who underwent immunotherapy or a combination of immunotherapy and chemotherapy. Future research should aim to expand the cohort to include patients treated with chemotherapy alone, analyzing whether the FIB-4 levels or liver fibrosis can specifically predict the efficacy of immunotherapy in cholangiocarcinoma. Furthermore, the tumor’s immune microenvironment plays a crucial role in influencing immune responses. Tertiary lymphoid structures can indicate the local immune infiltration status and their presence in various tumors has been associated with better immune responses (60, 61). Thus, they hold significant potential for predicting immune reactions in cholangiocarcinoma (62) and warrant further exploration in subsequent studies.

Future research should focus on finding clinically simple and accessible indicators to guide treatment and predict whether there is a benefit from treatment modalities, providing support for clinical decision-making and maximizing patient benefits with minimal cost.

## 5 Conclusion

Our study indicates that immune checkpoint inhibitors can control the progression of most cholangiocarcinoma, with side effects within a safe range. FIB-4 may serve as a prognostic predictor for patients with cholangiocarcinoma receiving immunotherapy.

## Data availability statement

The raw data supporting the conclusions of this article will be made available by the authors, without undue reservation.

## Ethics statement

The studies involving humans were approved by Research Ethics Committee of Tongji Hospital, Tongji Medical College, Huazhong University of Science and Technology. The studies were conducted in accordance with the local legislation and institutional requirements. The participants provided their written informed consent to participate in this study.

## Author contributions

ZZ: Conceptualization, Formal Analysis, Investigation, Methodology, Project administration, Writing – original draft, Writing – review & editing. JZ: Conceptualization, Data curation, Investigation, Writing – review & editing. MC: Conceptualization, Resources, Writing – review & editing. XH: Conceptualization, Software, Writing – review & editing. XG: Conceptualization, Supervision, Writing – review & editing. DZ: Conceptualization, Validation, Writing – review & editing. TG: Conceptualization, Visualization, Writing – review & editing. YY: Conceptualization, Funding acquisition, Writing – review & editing.

## References

[1] Lim JH Park CK. Pathology of cholangiocarcinoma. Abdom Imaging. (2004) 29:540–7. doi: 10.1007/s00261-004-0187-2 15383897

[2] Esnaola NF Meyer JE Karachristos A Maranki JL Camp ER Denlinger CS. Evaluation and management of intrahepatic and extrahepatic cholangiocarcinoma. Cancer. (2016) 122:1349–69. doi: 10.1002/cncr.29692 26799932

[3] Rushbrook SM Kendall TJ Zen Y Albazaz R Manoharan P Pereira SP. British Society of Gastroenterology guidelines for the diagnosis and management of cholangiocarcinoma. Gut. (2023) 73:16–46. doi: 10.1136/gutjnl-2023-330029 37770126 PMC10715509

[4] Buettner S Groot Koerkamp B. The Japanese Clinical Practice Guidelines for intrahepatic cholangiocarcinoma: a comparison with Western guidelines. Hepatobiliary Surg Nutr. (2023) 12:244–7. doi: 10.21037/hbsn PMC10129902 37124682

[5] Benson AB D'angelica MI Abrams T Abbott DE Ahmed A Anaya DA. NCCN guidelines(R) insights: biliary tract cancers, version 2.2023. J Natl Compr Canc Netw. (2023) 21:694–704. doi: 10.6004/jnccn.2023.0035 37433432

[6] Piha-Paul SA Oh DY Ueno M Malka D Chung HC Nagrial A. Efficacy and safety of pembrolizumab for the treatment of advanced biliary cancer: Results from the KEYNOTE-158 and KEYNOTE-028 studies. Int J Cancer. (2020) 147:2190–8. doi: 10.1002/ijc.33013 32359091

[7] Rimini M Fornaro L Lonardi S Niger M Lavacchi D Pressiani T. Durvalumab plus gemcitabine and cisplatin in advanced biliary tract cancer: An early exploratory analysis of real-world data. Liver Int. (2023) 43:1803–12. doi: 10.1016/j.annonc.2023.04.097 37452505

[8] Lei Z Ma W Si A. Effect of different PD-1 inhibitor combination therapies for unresectable intrahepatic cholangiocarcinoma. Aliment Pharmacol Ther. (2023) 58:611–22. doi: 10.1111/apt.17623 37349908

[9] Zhang Z Wang X Li H. Case Report: Camrelizumab combined with gemcitabine and oxaliplatin in the treatment of advanced intrahepatic cholangiocarcinoma: a case report and literature review. Front Immunol. (2023) 14:1230261. doi: 10.3389/fimmu.2023.1230261 37671157 PMC10475830

[10] Zhou N Li X Yang Y. Sintilimab plus nab-paclitaxel as second-line treatment for advanced biliary tract cancer: study protocol for an investigator-initiated phase 2 trial (NapaSinti trial). BMC Cancer. (2023) 23:729. doi: 10.1186/s12885-023-11188-4 37550655 PMC10405505

[11] Chao J Wang S Wang H. Real-world cohort study of PD-1 blockade plus lenvatinib for advanced intrahepatic cholangiocarcinoma: effectiveness, safety, and biomarker analysis. Cancer Immunol Immunother. (2023) 72:3717–26. doi: 10.1007/s00262-023-03523-2 PMC10991235 37787790

[12] Sterling RK Lissen E Clumeck N. Development of a simple noninvasive index to predict significant fibrosis in patients with HIV/HCV coinfection. Hepatology. (2006) 43:1317–25. doi: 10.1002/(ISSN)1527-3350 16729309

[13] Shah AG Lydecker A Murray K. Comparison of noninvasive markers of fibrosis in patients with nonalcoholic fatty liver disease. Clin Gastroenterol Hepatol. (2009) 7:1104–12. doi: 10.1016/j.cgh.2009.05.033 PMC3079239 19523535

[14] Kamada Y Munekage K Nakahara T. The FIB-4 index predicts the development of liver-related events, extrahepatic cancers, and coronary vascular disease in patients with NAFLD. Nutrients. (2022) 15. doi: 10.3390/nu15010066 PMC9824239 36615725

[15] Ke MY Xu T Fang Y. Liver fibrosis promotes immune escape in hepatocellular carcinoma via GOLM1-mediated PD-L1 upregulation. Cancer Lett. (2021) 513:14–25. doi: 10.1016/j.canlet.2021.05.007 33992711

[16] Aziz MH Sideras K Aziz NA Mauff K Haen R Roos D. The systemic-immune-inflammation index independently predicts survival and recurrence in resectable pancreatic cancer and its prognostic value depends on bilirubin levels: A retrospective multicenter cohort study. Ann Surg. (2019) 270(1):139–46. doi: 10.1097/SLA.0000000000002660 29334554

[17] Yi J Xue J Yang L Xia L He W. Predictive value of prognostic nutritional and systemic immune-inflammation indices for patients with microsatellite instability-high metastatic colorectal cancer receiving immunotherapy. Front Nutr. (2023) 10:1094189. doi: 10.3389/fnut.2023.1094189 37275637 PMC10232767

[18] Fornarini G Rebuzzi SE Banna GL Calabro F Scandurra G De Giorgi U. Immune-inflammatory biomarkers as prognostic factors for immunotherapy in pretreated advanced urinary tract cancer patients: an analysis of the Italian SAUL cohort. ESMO Open. (2021) 6(3):100118. doi: 10.1016/j.esmoop.2021.100118 33984678 PMC8134706

[19] Nenclares P Gunn L Soliman H Bover M Trinh A Leslie I. On-treatment immune prognostic score for patients with relapsed and/or metastatic head and neck squamous cell carcinoma treated with immunotherapy. J Immunother Cancer. (2021) 9(6). doi: 10.1136/jitc-2021-002718 PMC8190047 34103355

[20] Tsunematsu M Uwagawa T Onda S Shirai Y Okui N Matsumoto M. Systemic inflammation adversely affects response to anamorelin in patients with pancreatic cancer. Support Care Cancer. (2023) 31(12):732. doi: 10.1007/s00520-023-08206-3 38055066

[21] Li C Wu J Jiang L Zhang L Huang J Tian Y. The predictive value of inflammatory biomarkers for major pathological response in non-small cell lung cancer patients receiving neoadjuvant chemoimmunotherapy and its association with the immune-related tumor microenvironment: a multi-center study. Cancer Immunol Immunother. (2023) 72(3):783–94. doi: 10.1007/s00262-022-03262-w PMC10991885 36056951

[22] Jomrich G Paireder M Kristo I Baierl A Ilhan-Mutlu A Preusser M. High systemic immune-inflammation index is an adverse prognostic factor for patients with gastroesophageal adenocarcinoma. Ann Surg. (2021) 273(3):532–41. doi: 10.1097/SLA.0000000000003370 31425286

[23] Okadome K Baba Y Yagi T Kiyozumi Y Ishimoto T Iwatsuki M. Prognostic nutritional index, tumor-infiltrating lymphocytes, and prognosis in patients with esophageal cancer. Ann Surg. (2020) 271(4):693–700. doi: 10.1097/SLA.0000000000002985 30308614

[24] Hernando-Calvo A Mirallas O Marmolejo D Saavedra O Vieito M Assaf Pastrana JD. Nutritional status associates with immunotherapy clinical outcomes in recurrent or metastatic head and neck squamous cell carcinoma patients. Oral Oncol. (2023) 140:106364. doi: 10.1016/j.oraloncology.2023.106364 36989964

[25] Schwartz LH Seymour L Litiere S Ford R Gwyther S Mandrekar S. RECIST 1.1 - Standardisation and disease-specific adaptations: Perspectives from the RECIST Working Group. Eur J Cancer. (2016) 62:138–45. doi: 10.1016/j.ejca.2016.03.082 PMC5737786 27237360

[26] Shi GM Huang XY Wu D Sun HC Liang F Ji Y. Toripalimab combined with lenvatinib and GEMOX is a promising regimen as first-line treatment for advanced intrahepatic cholangiocarcinoma: a single-center, single-arm, phase 2 study. Signal Transduct Target Ther. (2023) 8:106. doi: 10.1038/s41392-023-01317-7 36928584 PMC10020443

[27] Elias C Zeidan YH Bouferraa Y Mukherji D Temraz S Charafeddine M. A phase II single arm study of Nivolumab with stereotactic Ablative radiation Therapy after induction chemotherapy in CHOlangiocarcinoma (NATCHO). BMC Cancer. (2022) 22(1):1296. doi: 10.1186/s12885-022-10373-1 36503610 PMC9743639

[28] ZhouQQGuoJWangZLiJChenMXuQ. Rapid visualization of PD-L1 expression level in glioblastoma immune microenvironment via machine learning cascade-based Raman histopathology. J Adv Res. (2023). doi: 10.1016/j.jare.2023.12.002 38072311

[29] Ye Z Zhang Y Chen J Wang X Hong Y Zhao Q. First-line PD-1 inhibitors combination therapy for patients with advanced cholangiocarcinoma: A retrospective real-world study. Int Immunopharmacol. (2023) 120:110344. doi: 10.1016/j.intimp.2023.110344 37245298

[30] Zhu C Li H Yang X Wang S Wang Y Zhang N. Efficacy, safety, and prognostic factors of PD-1 inhibitors combined with lenvatinib and Gemox chemotherapy as first-line treatment in advanced intrahepatic cholangiocarcinoma: a multicenter real-world study. Cancer Immunol Immunother. (2023) 72(9):2949–60. doi: 10.1007/s00262-023-03466-8 PMC10412480 37247023

[31] Zhang Y Wang X Li Y Hong Y Zhao Q Ye Z. Immune-related adverse events correlate with the efficacy of PD-1 inhibitors combination therapy in advanced cholangiocarcinoma patients: A retrospective cohort study. Front Immunol. (2023) 14:1141148. doi: 10.3389/fimmu.2023.1141148 37033935 PMC10079946

[32] Ma D Liu M Zhai X Li X Jin B Liu Y. Development and validation of prognostic risk prediction models for hepatocellular carcinoma patients treated with immune checkpoint inhibitors based on a systematic review and meta-analysis of 47 cohorts. Front Immunol. (2023) 14:1215745. doi: 10.3389/fimmu.2023.1215745 37520554 PMC10380940

[33] Chen S Han L Guo S Tan Z Dai G. Hyperprogressive disease during PD-1 blockade in patients with advanced pancreatic cancer. Hum Vaccin Immunother. (2023) 19:2252692. doi: 10.1080/21645515.2023.2252692 37675466 PMC10486295

[34] Ratti F Marino R Olthof PB Pratschke J Erdmann JI Neumann UP. Predicting futility of upfront surgery in perihilar cholangiocarcinoma: Machine learning analytics model to optimize treatment allocation. Hepatology. (2024) 79:341–54. doi: 10.1097/HEP.0000000000000554 37530544

[35] Zhang H Li Q Huang G Yang Z Chen K Meng B. Construction and validation of a novel prognostic model for intrahepatic cholangiocarcinoma based on a combined scoring system of systemic immune-inflammation index and albumin-bilirubin: a multicenter study. Front Oncol. (2023) 13:1239375. doi: 10.3389/fonc.2023.1239375 37841429 PMC10569214

[36] Zhu J Wang D Liu C Huang R Gao F Feng X. Development and validation of a new prognostic immune-inflammatory-nutritional score for predicting outcomes after curative resection for intrahepatic cholangiocarcinoma: A multicenter study. Front Immunol. (2023) 14:1165510. doi: 10.3389/fimmu.2023.1165510 37063918 PMC10102611

[37] Rigor J Diegues A Presa J Barata P Martins-Mendes D. Noninvasive fibrosis tools in NAFLD: validation of APRI, BARD, FIB-4, NAFLD fibrosis score, and Hepamet fibrosis score in a Portuguese population. Postgrad Med. (2022) 134(4):435–40. doi: 10.1080/00325481.2022.2058285 35332833

[38] Younes R Caviglia GP Govaere O Rosso C Armandi A Sanavia T. Long-term outcomes and predictive ability of non-invasive scoring systems in patients with non-alcoholic fatty liver disease. J Hepatol. (2021) 75(4):786–94. doi: 10.1016/j.jhep.2021.05.008 34090928

[39] Liao R Li DW Du CY Li M. Combined preoperative ALBI and FIB-4 is associated with recurrence of hepatocellular carcinoma after curative hepatectomy. J Gastrointest Surg. (2018) 22(10):1679–87. doi: 10.1007/s11605-018-3810-1 29777455

[40] Loosen SH Kostev K Demir M Luedde M Keitel V Luedde T. An elevated FIB-4 score is associated with an increased incidence of liver cancer: A longitudinal analysis among 248,224 outpatients in Germany. Eur J Cancer. (2022) 168:41–50. doi: 10.1016/j.ejca.2022.03.010 35436676

[41] Loosen SH Kostev K Keitel V Tacke F Roderburg C Luedde T. An elevated FIB-4 score predicts liver cancer development: A longitudinal analysis from 29,999 patients with NAFLD. J Hepatol. (2022) 76(1):247–8. doi: 10.1016/j.jhep.2021.08.030 34520785

[42] Unalp-Arida A Ruhl CE. Liver fibrosis scores predict liver disease mortality in the United States population. Hepatology. (2017) 66:84–95. doi: 10.1002/hep.29113 28195363 PMC7005915

[43] Sung KC Johnston MP Lee MY Byrne CD. Non-invasive liver fibrosis scores are strongly associated with liver cancer mortality in general population without liver disease. Liver Int. (2020) 40(6):1303–15. doi: 10.1111/liv.14416 32090451

[44] Suh B Yun JM Park S Shin DW Lee TH Yang HK. Prediction of future hepatocellular carcinoma incidence in moderate to heavy alcohol drinkers with the FIB-4 liver fibrosis index. Cancer. (2015) 121(21):3818–25. doi: 10.1002/cncr.29577 26178294

[45] Ampuero J Carmona I Sousa F Rosales JM Lopez-Garrido A Casado M. A 2-step strategy combining FIB-4 with transient elastography and ultrasound predicted liver cancer after HCV cure. Am J Gastroenterol. (2022) 117(1):138–46. doi: 10.14309/ajg.0000000000001503 34817975

[46] Ioannou GN Beste LA Green PK Singal AG Tapper EB Waljee AK. Increased risk for hepatocellular carcinoma persists up to 10 years after HCV eradication in patients with baseline cirrhosis or high FIB-4 scores. Gastroenterology. (2019) 157(5):1264–1278 e4. doi: 10.1053/j.gastro.2019.07.033 31356807 PMC6815714

[47] John BV Dang Y Kaplan DE Jou JH Taddei TH Spector SA. Liver stiffness measurement and risk prediction of hepatocellular carcinoma after HCV eradication in veterans with cirrhosis. Clin Gastroenterol Hepatol. (2024) 22(4):778–788 e7. doi: 10.1016/j.cgh.2023.11.020 38061410 PMC10960676

[48] Tai J Hsu CW Chen WT Yang SS Chiu CH Chien RN. Association of liver fibrosis with extrahepatic cancer in steatotic liver disease patients with PNPLA3 I148M GG genotype. Cancer Sci. (2024) 115(2):564–74. doi: 10.1111/cas.16042 PMC10859614 38083881

[49] Kim GA Lee HC Choe J Kim MJ Lee MJ Chang HS. Association between non-alcoholic fatty liver disease and cancer incidence rate. J Hepatol. (2017). doi: 10.1016/j.jhep.2017.09.012 29150142

[50] Akiyama T Miyamoto Y Imai K Yamashita Y Nomoto D Daitoku N. Fibrosis-4 index, a noninvasive fibrosis marker, predicts survival outcomes after hepatectomy for colorectal cancer liver metastases. Ann Surg Oncol. (2020) 27(9):3534–41. doi: 10.1245/s10434-020-08828-5 32648180

[51] Lv Y Zhang HJ. Effect of non-alcoholic fatty liver disease on the risk of synchronous liver metastasis: analysis of 451 consecutive patients of newly diagnosed colorectal cancer. Front Oncol. (2020) 10:251. doi: 10.3389/fonc.2020.00251 32181157 PMC7059642

[52] Xu K Shi M Zhang W Shi Y Dong Q Shen X. Preoperative fibrosis-4 (FIB-4) evaluation may be helpful to evaluate prognosis of gastric cancer patients undergoing operation: A retrospective study. Front Oncol. (2021) 11:655343. doi: 10.3389/fonc.2021.655343 34221972 PMC8247641

[53] Seo YG Polyzos SA Park KH Mantzoros CS. Fibrosis-4 index predicts long-term all-cause, cardiovascular and liver-related mortality in the adult Korean population. Clin Gastroenterol Hepatol. (2023) 21(13):3322–35. doi: 10.1016/j.cgh.2023.04.026 37164111

[54] Sutandyo N Kurniawati SA Jayusman AM Syafiyah AH Pranata R Hanafi AR. Repurposing FIB-4 index as a predictor of mortality in patients with hematological Malignancies and COVID-19. PloS One. (2021) 16(9):e0257775. doi: 10.1371/journal.pone.0257775 34555104 PMC8459998

[55] Miele L Dajko M Savino MC Capocchiano ND Calvez V Liguori A. Fib-4 score is able to predict intra-hospital mortality in 4 different SARS-COV2 waves. Intern Emerg Med. (2023) 18(5):1415–27. doi: 10.1007/s11739-023-03310-y PMC10412472 37491564

[56] Zheng F Meng Q Zhang L Chen J Zhao L Zhou Z. Prognostic roles of hematological indicators for the efficacy and prognosis of immune checkpoint inhibitors in patients with advanced tumors: a retrospective cohort study. World J Surg Oncol. (2023) 21(1):198. doi: 10.1186/s12957-023-03077-8 37420219 PMC10326931

[57] Li X Wang Y Zhai Z Mao Q Chen D Xiao L. Predicting response to immunotherapy in gastric cancer via assessing perineural invasion-mediated inflammation in tumor microenvironment. J Exp Clin Cancer Res. (2023) 42(1):206. doi: 10.1186/s13046-023-02730-0 37563649 PMC10416472

[58] Hu B Yang XR Xu Y Sun YF Sun C Guo W. Systemic immune-inflammation index predicts prognosis of patients after curative resection for hepatocellular carcinoma. Clin Cancer Res. (2014) 20(23):6212–22. doi: 10.1158/1078-0432.CCR-14-0442 25271081

[59] Zhang CL Jiang XC Li Y Pan X Gao MQ Chen Y. Independent predictive value of blood inflammatory composite markers in ovarian cancer: recent clinical evidence and perspective focusing on NLR and PLR. J Ovarian Res. (2023) 16(1):36. doi: 10.1186/s13048-023-01116-2 36759864 PMC9912515

[60] Cabrita R Lauss M Sanna A Donia M Skaarup Larsen M Mitra S. Tertiary lymphoid structures improve immunotherapy and survival in melanoma. Nature. (2020) 577(7791):561–5. doi: 10.1038/s41586-019-1914-8 31942071

[61] Wang B Liu J Han Y Deng Y Li J Jiang Y. The presence of tertiary lymphoid structures provides new insight into the clinicopathological features and prognosis of patients with breast cancer. Front Immunol. (2022) 13:868155. doi: 10.3389/fimmu.2022.868155 35664009 PMC9161084

[62] Shang T Jiang T Lu T Wang H Cui X Pan Y. Tertiary lymphoid structures predict the prognosis and immunotherapy response of cholangiocarcinoma. Front Immunol. (2023) 14:1166497. doi: 10.3389/fimmu.2023.1166497 37234171 PMC10206168

