# Supplementary material for: The fibrosis-4 index is a prognostic factor for cholangiocarcinoma patients who received immunotherapy
Source: Front Immunol. 2024 May 10;15:1376590. doi: 10.3389/fimmu.2024.1376590 (PMC11116781; doi:10.3389/fimmu.2024.1376590)
Supplement: Supplementary file 1 [file DataSheet_1.docx]

Supplementary Material


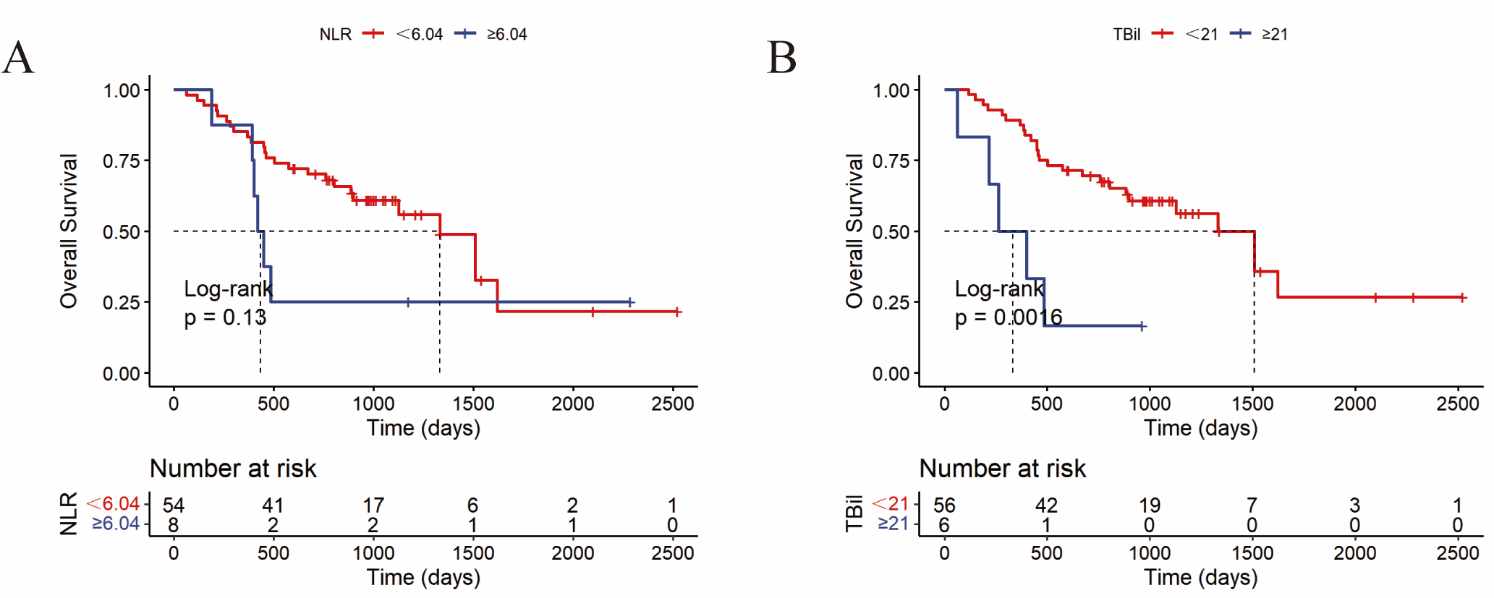


Fig. S1. Kaplan-Meier analyses of NLR (A) and TBil (B) with OS as the outcome variable.
